# Natural flavonols: actions, mechanisms, and potential therapeutic utility for various diseases

**DOI:** 10.1186/s43088-023-00387-4

**Published:** 2023-05-15

**Authors:** Aar Rafi Mahmud, Tanzila Ismail Ema, Mohd. Faijanur - Rob Siddiquee, Asif Shahriar, Hossain Ahmed, Md. Mosfeq-Ul-Hasan, Nova Rahman, Rahatul Islam, Muhammad Ramiz Uddin, Md. Furkanur Rahaman Mizan

**Affiliations:** 1 Department of Biochemistry and Molecular Biology, Mawlana Bhashani Science and Technology University, Santosh, Tangail, 1902 Bangladesh; 2 Department of Biochemistry and Microbiology, North South University, Dhaka, 1229 Bangladesh; 3 Department of Biochemistry and Molecular Biology, University of Dhaka, Dhaka, 1000 Bangladesh; 4 Department of Microbiology, Stamford University Bangladesh, 51 Siddeswari Road, Dhaka, 1217 Bangladesh; 5 Department of Biotechnology and Genetic Engineering, University of Development Alternative (UODA), Dhaka, 1208 Bangladesh; 6 Hajee Mohammad Danesh Science and Technology University, Dinajpur, 5200 Bangladesh; 7 Department of Biochemistry and Molecular Biology, Jahangirnagar University, Savar, Dhaka, 1342 Bangladesh; 8 Department of Genetic Engineering and Biotechnology, Shahjalal University of Science and Technology, Sylhet, Bangladesh; 9 Department of Pharmacy, Jahangirnagar University, Savar, Dhaka, Bangladesh; 10 Department of Food Science and Technology, Chung-Ang University, Anseong, South Korea

**Keywords:** Flavonol, Quercetin, Myricetin, Kaempferol, Antioxidant

## Abstract

**Background:**

Flavonols are phytoconstituents of biological and medicinal importance. In addition to functioning as antioxidants, flavonols may play a role in antagonizing diabetes, cancer, cardiovascular disease, and viral and bacterial diseases. Quercetin, myricetin, kaempferol, and fisetin are the major dietary flavonols. Quercetin is a potent scavenger of free radicals, providing protection from free radical damage and oxidation-associated diseases.

**Main body of the abstract:**

An extensive literature review of specific databases (e.g., Pubmed, google scholar, science direct) were conducted using the keywords “flavonol,” “quercetin,” “antidiabetic,” “antiviral,” “anticancer,” and “myricetin.” Some studies concluded that quercetin is a promising antioxidant agent while kaempferol could be effective against human gastric cancer. In addition, kaempferol prevents apoptosis of pancreatic beta-cells via boosting the function and survival rate of the beta-cells, leading to increased insulin secretion. Flavonols also show potential as alternatives to conventional antibiotics, restricting viral infection by antagonizing the envelope proteins to block viral entry.

**Short conclusion:**

There is substantial scientific evidence that high consumption of flavonols is associated with reduced risk of cancer and coronary diseases, free radical damage alleviation, tumor growth prevention, and insulin secretion improvement, among other diverse health benefits. Nevertheless, more studies are required to determine the appropriate dietary concentration, dose, and type of flavonol for a particular condition to prevent any adverse side effects.

## Background

**Fig. 1:**
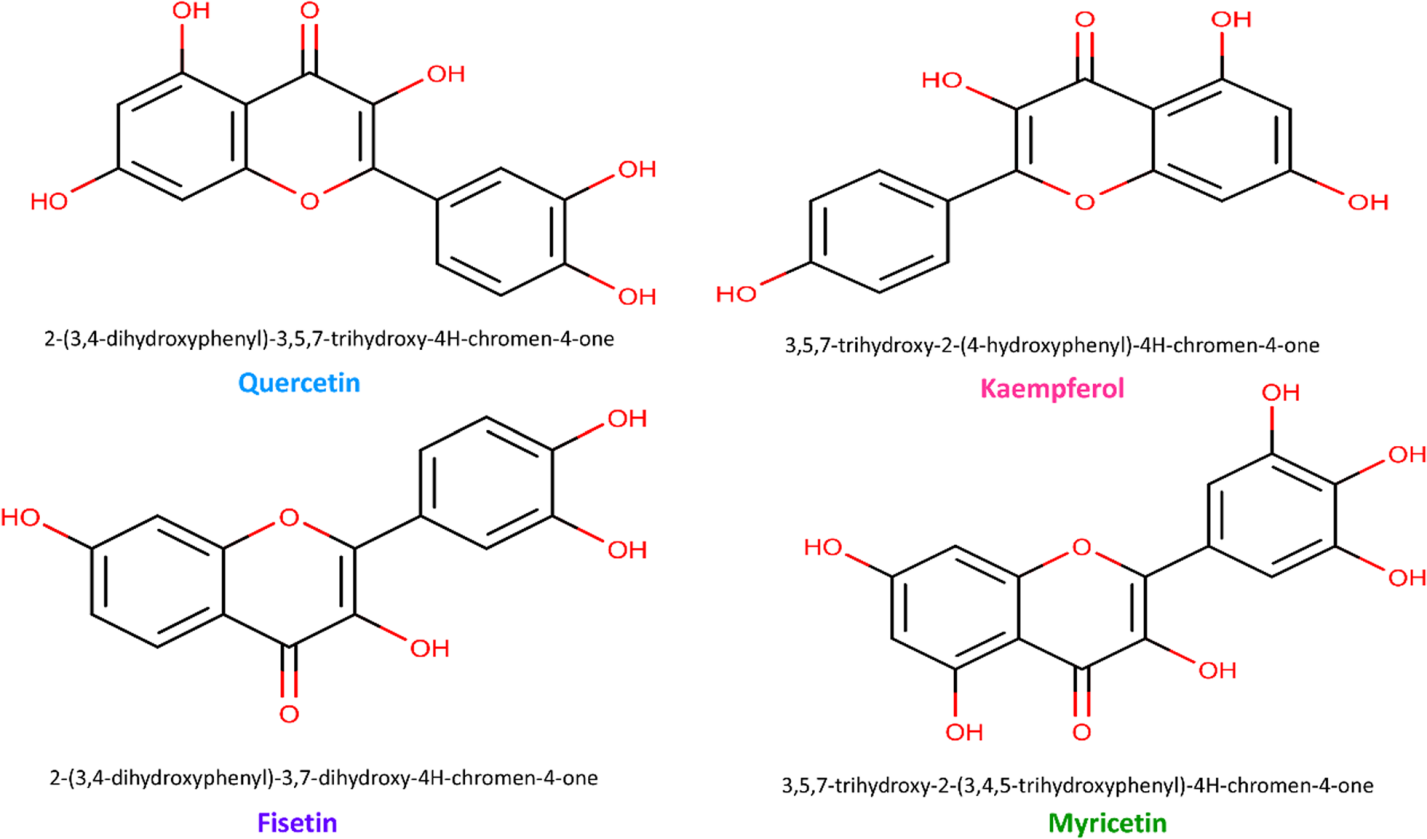
Structural formula of some promising medicinal flavanols compound

Flavonols are polyphenols that are especially abundant in broccoli, apples, grapes, tomatoes, onion, kale, broccoli, tea, red wine, olive, and citrus fruits. The structure of flavonols is represented by the C6–C3–C6 model in which two benzene rings are joined by a linear three-carbon chain (C2, C3, and C4) with a double bond between C2 and C3 and a carbonyl moiety on C4 (Fig. 1) [77, 167]. Structure–activity studies indicate that the number and arrangement of hydroxyl moieties in flavonols are important determinants of their antioxidant and biological activities [77, 86].

Flavonols act as antioxidants against a diverse range of stressors. Their photoprotective action is well reported [29, 127], yet how the biosynthesis of flavonols is regulated by ultraviolet (UV) light, the significance of their role in photoprotection, and whether the reactive oxygen species (ROS)-scavenging action of flavonols acts in concert with other signaling pathways in UV protection *in planta* remain unknown [55, 164]. Nonetheless, it is well established that flavonols control critical psychological functions in multicellular organisms when their redox balance is impaired by natural factors [162]. The transcriptional regulation of flavonol biosynthesis in plants is mediated by myeloblastosis (MYB) transcription factors driven by oxidation–reduction reaction; interestingly, the protective roles given to flavonols outweigh their ability to minimize various sources of reactive oxygen [121]. In addition to contributing to stress resistance in plants, flavonols behave as regulators of maturation and differentiation during plant development due to their affinity for a myriad of proteins involved in signaling pathways critical to cell growth and development [121, 149].

Plants universally increase their flavonol concentrations in response to environmental challenges, including high sunlight intensity, drought, and pathogen attack, creating an efficient strategy to regulate stress-induced ROS production [56, 87, 147]. Previous studies suggest that flavonol accumulation in plants may be induced by auxin and ethylene, key hormones that regulate plant growth and development, indicating associations between the auxin and ethylene signaling pathways and flavonoid regulatory and structural target genes [17, 89]. For example, in *Arabidopsis*, ethylene-induced flavonol production alleviates ROS in guard cells and affects stomatal conductance [141, 159]. Plant guard cells are essential for photosynthesis and transpiration, regulating the size of the tiny pores or stomata on the surface of leaves in response to environmental cues, such as water status, carbon dioxide concentration, calcium (Ca^2+^), and ROS.

Oxidative stress has been attributed to inflammation, atherosclerosis, ischemic damage, cancer, and neurodegenerative disorders, like Parkinson’s and Alzheimer’s. Myricetin, fisetin, quercetin, and kaempferol are the most common flavonol aglycones in fruits and vegetables and show high diversity in their methylation, hydroxylation, and glycosylation patterns [137].

**Fig. 2:**
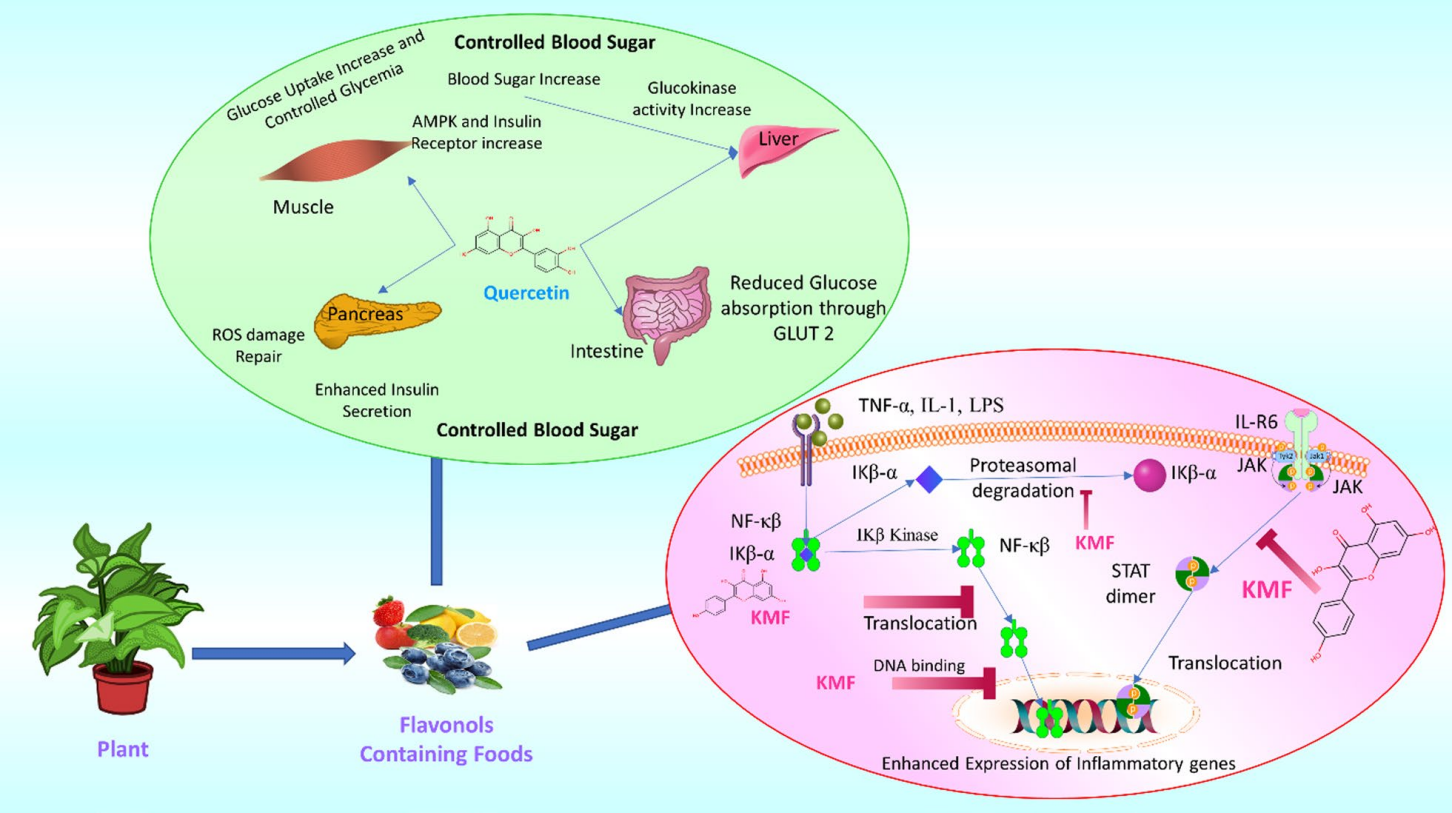
Plant-derived flavonol-containing foods have shown profound medicinal activity against disease and maintaining a healthy diet. Quercetin is found to be useful in homeostasis and as a modulator of controlled blood sugar levels in the body. Associate transporters and organs with enzymatic activity are regulated by quercetin, which controls the organ systems by either increasing or decreasing enzymatic activity, enzyme secretion, and expression of transporters, signaling pathways, and other aspects. It controls ROS (Reactive oxygen species) production in the absence of glucose in the pancreatic cell. Besides, quercetin also alleviates glucose assimilation via the GLUT2 receptor. Kaempferol (KMF) is observed in the anti-inflammatory response of cells against pathogen-mediated inflammation. Inflammation is a natural phenomenon that protects the host from damage by pathogens. Uncontrolled inflammation generates a cytokine storm that leads to potential damage to cells. Kaempferol inhibits many inflammatory signal transduction pathways at various levels (i.e., NF-kB, JAK-STAT pathway) NF-kB = Nuclear Factor kB

The hydroxyl group in the third position of the regular flavone structure is the key structural feature responsible for the antioxidant and biological action of flavonols. Flavonols are well known as antioxidants that help in scavenging free radicals that could cause serious heart diseases and cancer growth. Quercetin is used as a representative flavonol to illustrate the mechanistic action of scavenging toxic radicals, protecting the body from serious ailments (Fig. 2).

The perspective of using flavonols as pharmacophores is very intriguing and convincing because of their multipurpose therapeutic effects. However, low bioavailability and bioequivalence have impeded the large-scale production of flavonols as commercial “drugs.” Nanotechnology, such as nanotool delivery systems, may bridge this gap. However, along with this advanced technology, other factors have to be considered, such as the effective route of flavonol administration, the dose of the flavonols, suitability to patients, and the type and process of nanoparticle delivery system [61]. The biological pathways and associated functions of natural flavonols as medicinal compounds for treating various diseases will be illustrated in this review.

## Main text

### Classification of flavonols

Flavonols are a subclass of flavonoids characterized by a 3-hydroxyflavone backbone. In higher plants, flavonols are typically present in glycosylated forms, preferentially at C3 as mono-, di-, or triglycosides. The sugar residues commonly found in flavonols are glucose, galactose, rhamnose, and glucuronic acid. Flavonols can be classified based on their mechanistic actions.

### Therapeutic aspects of flavonols

Research suggests that high consumption of flavonols can alleviate cancer, cardiovascular disease, diabetes, viral infections, bacterial infections, and other disease-related mortality threats [14, 27, 32, 150, 177]. For example, various mechanisms of flavonol action on the androgen receptor (AR)-mediated signaling cascades have been proposed for their chemopreventive action in castrate-resistant prostate cancer [14], and quercetin has undisputed endothelium-independent vasodilator actions, prevents endothelial dysfunction, and prevents myocardial ischemic injury [116] and it is well recognized for its antioxidant function, and it inhibits the action of a large variety of enzymes. As a result, quercetin is expected to impair a range of metabolic signaling mechanisms and biological and clinical processes. In the context of diabetes, there is mounting evidence that flavonols may influence carbohydrate metabolism by modulating α-amylase (the glucose-generating digestive enzyme) and the secondary active transporters (sodium-glucose cotransporter 1 [SGLT1] and glucose transporter 2 [GLUT2]) that mediate intestinal glucose absorption, among various other proposed mechanisms [53, 163]. Moreover, the use of flavonols for the treatment of various infectious diseases is associated with their ability to penetrate the bacterial cell membrane and interact with essential enzymes (e.g., DNA gyrase, DNA PriA helicase, ATPase) [1, 50, 58, 60, 169], including enzymes critical for viral replication, transcription, or integration into host cells (e.g., phosphatidylinositol 3-kinase, RNA polymerase, and 3C-like protease [3CL^pro^], a cysteine protease) [3, 106].

There is mounting evidence that medicinal plants have a large pool of antiviral compounds that can be developed into potential drug and vaccine candidates after further assessment and research via laboratory experiments and in silico investigations. These plants possess the structural diversity and strong adapting tendency to survive in changing environmental conditions, proving them to be a vital and sustainable source for creating potent drugs and vaccines [122]. Table 1 represents the most common flavonols pharmaceuticals properties with their natural sources.

**Table 1:** Natural sources and pharmaceutical properties of flavonols

Name	Pharmaceutical’s properties	Natural sources	Reference
Apigenin	Antioxidant, anti-inflammatory, and hepatoprotective	Chamomile, parsley, onions, oranges, wheat sprouts	[176]
Catechin	Antioxidant, anti-inflammatory, anticancer	Green tea, cocoa, apples, grapes, berries, and other fruits and vegetables	[11, 78, 103, 114]
Epicatechin	Antioxidant, anti-inflammatory, anticancer	Cocoa, tea, apples, grapes, berries, and other fruits and vegetables	[11, 20, 54]
Eriodictyol	Antioxidant, anti-inflammatory, anticancer, antiviral, and cardioprotective	Citrus fruits, parsley, and other fruits and vegetables	[35]
Fisetin	Antioxidant, anti-inflammatory, anticancer, neuroprotective, and cardioprotective	Strawberries, apples, persimmons, grapes, onions, cucumbers, and other fruits and vegetables	[83, 182, 183]
Hesperetin	Antioxidant, anti-inflammatory, anticancer, antiviral, neuroprotective, and cardioprotective	Citrus fruits, especially oranges	[129, 158, 182, 183]
Kaempferol	Antioxidant, anti-inflammatory, anticancer, antidiabetic, antimalarial, hepatoprotective neuroprotective, and cardioprotective	Tea, broccoli, kale, spinach, beans, berries, and other fruits and vegetables	[6, 119]
Myricetin	Antioxidant, anti-inflammatory, anticancer, antidiabetic, antimalarial, neuroprotective, and cardioprotective	Berries, grapes, onions, tea, and other fruits and vegetables	[90, 91, 143, 146]
Naringenin	Antioxidant, anti-inflammatory, anticancer, antidiabetic, neuroprotective, and cardioprotective	Citrus fruits, especially grapefruits	[9, 130]
Pinocembrin	Antioxidant, anti-inflammatory, anticancer, neuroprotective, and cardioprotective	Propolis, honey, and some fruits and vegetables	[39]
Procyanidins	Antioxidant, anti-inflammatory, anticancer	Cocoa, red wine, apples, grapes, berries, and other fruits and vegetables	[26]
Quercetin	Antioxidant, anti-inflammatory, anticancer, antiviral, anti-hypertension, antimalarial, neuroprotective, hepatoprotective and cardioprotective	Onions, apples, grapes, berries, broccoli, tea, red wine, and many other fruits and vegetables	[7, 40, 134]
Rutin	Antioxidant, anti-inflammatory, anticancer, antimalarial, neuroprotective, and cardioprotective	Buckwheat, asparagus, citrus fruits, and other fruits and vegetables	[46, 113, 131, 174]
Silymarin	Antioxidant, anti-inflammatory, anti-fibrotic, and hepatoprotective	Milk thistle	[2, 102]
Taxifolin	Antioxidant, anti-inflammatory, anticancer, antidiabetic, neuroprotective, and cardioprotective	Milk thistle, onions, citrus fruits, and other fruits and vegetables	[133, 151, 152]
Theaflavins	Antioxidant, anti-inflammatory, anticancer, antidiabetic, neuroprotective, and cardioprotective	Black tea	[88]
Thearubigin	Antioxidant, anti-inflammatory, anti tumor, anti-hypertensive	Black tea	[95]

### Anticancer activity of flavonols

The four most studied flavonols (kaempferol, quercetin, fisetin, and myricetin) associated with anticancer functionality are found in foods like olives, onions, various berries, and broccoli [14, 79]. Inhibition of 5-alpha reductase enzymes, interference with androgen and the androgen signaling axis, and suppression of the expression and activity of the AR complex by transactivation of coregulators specificity protein 1 (Sp1) and c-Jun transcription factors along with the phosphoinositide 3-kinase/ Ak strain transforming (PI3K/Akt) pathway are among various mechanisms underlying the chemopreventive effects of flavonols in prostate cancer [14]. Although cancer many react to androgen removal via operation and chemotherapy, the effect is temporary, with disease recurrence, eventually progressing to castrate-resistant prostate cancer, the lethal phenotype of the disease [16]. Tight regulation of the AR signaling homeostasis is critical to maintaining diverse cell functions because dysregulation of this homeostasis leads to aberrant androgen responses and promotes prostate cancer [13]. Some indicators of AR action are prostate-specific antigen (PSA), human protein kallikrein-2 (HK2), and 5α-reductase enzyme [14, 44, 49]. Transcriptional measurements in vitro show that quercetin inhibits the production of PSA and HK2 by suppressing AR function [170, 175]. Kaempferol (extracted from pomegranate extract) hinders dihydrotestosterone-stimulated AR deposition in the nucleus and the expression of PSA and 5α-reductase in vitro [48]. Fisetin, a flavonol found in apple, persimmon, strawberry, onion, kiwifruit, and cucumber, competes with androgen to specifically bind to the ligand-binding domain of AR in lymph node carcinoma of the prostate (LNCaP) cell line. This interaction decreases AR stability and amino-terminal/carboxyl-terminal (N–C) interaction of AR, thereby thwarting transactivation of AR target genes and downregulating AR protein levels. Moreover, in AR-positive CWR22rv1 prostate cancer cell-bearing mice, fisetin inhibits tumor growth and decreases serum PSA levels [73].

The extracellular signal-regulated kinase (ERK) signaling pathway is essential for cancer progression and programmed cell death [19]. By lowering B-cell lymphoma 2 (Bcl-2) expression and causing mitochondrial membrane potential (MMP) impairment, the total ERK1/2 protein expression and phosphorylated ERK functions are inhibited, causing HeLa cervical cancer cells to die [24]. Kaempferol and myricetin have been shown to exert anticancer properties by alleviating phosphorylated ERK activity rather than total ERK protein expression [25, 68, 85]. The ERK signals are also the molecular targets of quercetin in the prevention and treatment of cancer [76].

Nevertheless, the exact functional mechanism by which flavonols reduce the total ERK protein expression in HeLa cells is yet to be determined [15]. In an assessment of the cytotoxic effects of kaempferol, myricetin, and quercetin in rhesus monkey kidney epithelial cells- Lilly Laboratories Culture-Monkey Kidney 2 (LLC-MK2), 5 and 10 μM myricetin and 50 µM quercetin separately hindered normal epithelial cells by cytotoxic assays based on 3-(4,5-dimethylthiazol-2-yl)-2,5-diphenyltetrazolium bromide (MTT) and sulforhodamine B, respectively, whereas kaempferol had no impact. However, in HeLa cells, kaempferol and quercetin, but not myricetin, showed marked cytotoxic activities on cancer cells and suppressed total ERK1/2 protein expression, with quercetin showing greater efficiency than kaempferol (Fig. 2) [15].

Fisetin induces apoptotic cell death in oral squamous cell carcinoma cell lines (Ca9-22 and CAL-27) via the mitochondrial pathway [110]. Notably, CAL-27 cells were more susceptible to fisetin, having a 50% inhibitory concentration (IC_50_) of 50 µM compared to 200 µM for Ca9-22 cells after incubation for 48 h [82]. Fisetin also induces apoptosis in several human laryngeal cancer cell lines (TU212, M2e, and Hep-2) via inhibiting tumor cell proliferation, inducing apoptosis and autophagy regulated by ERK1/2 and AKT/nuclear factor-kappa B (NF-κB)/mammalian target of rapamycin (mTOR) signaling pathways, with an IC_50_ of 10 µM [180]. In addition, chromatin contraction and nucleus shrinkage lead to programmed cell death and karyorrhexis (nucleus fragmentation), which occurred in oral squamous carcinoma cell lines (HSC-3, Ca9-22, and CAL-27) following fisetin treatment (B. S. [110, 139, 165]. The cytotoxic effects of fisetin also induce necrosis of TU212 cells [82].

Bcl-2 proteins, which include pro-apoptotic pore formers (BAX, BAK, and BOK) and pro-apoptotic BH3-only proteins (e.g., BAD, BID, BIK, BIM, BMF, HRK, NOXA, PUMA) as well as anti-apoptotic factors (e.g., BCL-2, BCL-XL, BCL-W, MCL-1, and BFL-1/A1), are key regulators of the intrinsic apoptotic pathway. Fisetin enhances the activity of pro-apoptotic proteins (e.g., BAD, BAX, NOXA, BOK) and caspase-3, caspase-8, and caspase-9 in head and neck cancer cells but alleviates the activity of anti-apoptotic BCL-2, MCL-1, XIAP, and BCL-X [82]. Moreover, fisetin escalates ROS levels in some oral cancer cell lines (HSC3 and SCC-4) to increase Ca^2+^ release and decrease the MMP, promoting programmed cell death [139, 144].

Kaempferol dose-dependently inhibits the growth of FaDu pharyngeal cancer cells, with 0.1 and 1 µM kaempferol [64, 64, 65, 65] showing a more potent effect than apigenin, a flavone found in tea [82]. Kaempferol also induces caspase-3-dependent apoptosis in SCC-1483 cells at various concentration doses [71]. In SSC-4 cells, kaempferol downregulates the ERK1/2 signaling pathway and inhibits activator protein-1 (AP-1) activity, markedly decreasing matrix metalloprotein-2 (MMP-2) expression, subsequently producing an antimetastatic effect [92]. The anticancer potency of kaempferol was further confirmed in a mice xenograft model, revealing the ability to significantly prevent the growth of tumor size coupled with a marked decrease in hexokinase-2 expression and epidermal growth factor receptor (EGFR) activity in tumor tissues [64, 65, 172].

### Flavonols against cardiovascular disease

The vascular endothelium precisely regulates cardiovascular homeostatic mechanisms. To maintain cardiovascular conditions, the balance among antithrombotic and prothrombotic determinants, antiproliferative and proliferative determinants, and vasoconstrictors and vasodilators should be maintained. If not, platelet accumulation, atherosclerosis, hypertension, and myocardial infarction may occur. Consequently, compromised endothelium-dependent vasodilation is characterized by decreased nitric oxide (NO) activity and the conversion of a normal antithrombotic endothelial state into a prothrombotic and pro-inflammatory state [116]. In most coronary diseases, endothelial malfunction is an initial and independent predictor of poor prognosis [132, 160]. Thus, high blood pressure, arteriosclerosis, myocardial infarction, and various other disorders have been linked to changes in endothelial activity.

Cardiovascular dysfunction is considered one of the major threats for human health, which leads to cardiac diseases and vascular dysfunction. The loss of the regulatory role of the vascular endothelium acts as a diagnostic method to identify early signs of diabetes-induced vascular dysfunction. Flavonols have shown promising therapeutic effects as vasoprotective agents by acting as vasorelaxants [38]. In a study of healthy male subjects, acute consumption of quercetin improved endothelial function by augmenting endogenous NO (S-nitrosothiols, nitrite, and nitrate) and reducing endothelin-1 production [99]. Conversely, in the endothelium-independent process, flavonols prevent extracellular Ca^2+^ influx by blocking the voltage-gated or receptor-gated Ca^2+^ channels, hampering the release of Ca^2+^ from the sarcoplasmic reticulum channels thereby arresting contraction of the vascular endothelium, causing it to relax. Furthermore, quercetin has exhibited activating effects on BKCa by a hydrogen peroxide-dependent process in coronary arteries of rat models [86].

Quercetin’s impact on endothelial development and impairment has been extensively researched [117]. Studies have shown that quercetin exerts systemic and coronary vasodilator effects in vitro and reduces cardiac hypertrophy, endothelial dysfunction, vascular remodeling, oxidative status, and blood pressure in a number of rat models of hypertension [62, 63, 118]. Depending on the experimental conditions, quercetin may also act as a pro-oxidant and generate ROS [30]. However, quercetin (as the aglycone) is usually not present in plasma, but it is rapidly methylated, glucuronidated, and sulfated during absorption in vivo. Quercetin’s effect on NO is often quite complicated, and oxidative pressure markedly impacts the result. In the presence of oxygen, quercetin can auto-oxidize and produce superoxide (O_2_^−^) within an in vitro framework that efficiently interacts with and neutralizes NO, an effect that is not observed for the sulfated and glucuronidated derivatives [94, 96]. Amperometric measurements showed that quercetin might elevate the NO level if there is no oxidative pressure [148]. Quercetin has also been shown to alleviate endothelial dysfunction by increasing NO synthesis involving large-conductance Ca^2+^-activated K^+^ channel (BKCa)-dependent membrane hyperpolarization-induced capacitative Ca^2+^ entry [75, 148].

Flavonols can interfere with many biochemical factors implicated in the pathobiology of myocardial infarction and cardiovascular disease, as this section illustrated. Such multiple associations are often beneficial, but they can also be harmful [116].

### Flavonols as antidiabetic agents

Diabetes mellitus is a major global health concern. It is defined as a chronic metabolic disorder characterized by the inability to maintain glucose homeostasis due to a malfunctioned glucose uptake mechanism. Diabetes mellitus is subdivided into type 1 diabetes mellitus (T1DM), which is classified as the absolute lack of insulin secretion due to the destruction of the beta-pancreatic cells, and type 2 diabetes mellitus (T2DM), in which insufficient insulin release occurs or the cells have developed resistance to insulin [153]. In addition, there is a phenomenon called “double diabetes” known for its persistent and unmanageable hyperglycemia, in which even a high dose of insulin fails to alleviate the blood glucose concentration. Ultimately, both T1DM and T2DM cause severe macrovascular dysfunction (cardiovascular disease, stroke) and microvascular dysfunction (nephropathy, neuropathy, retinal impairment) [61]. A clinical study has shown that long-term uncontrolled diabetes mellitus can also increase the risk of developing Alzheimer’s disease [4, 59]. Many therapeutic companies have developed drugs for the management of diabetes mellitus. However, the conventional methods have not generated the desired outcomes in patients, which have led scientists to study the reservoir of phytochemicals, more specifically, flavonols, as an alternative approach [38].

A growing body of evidence has exhibited an inverse correlation between the dietary intake of flavonols and the risk of T2DM. As already mentioned in this review, the antioxidant activity of flavonols can regulate the redox status and prevent damage caused by oxidative stress. Hyperglycemia causes increased ROS production due to the altered and high mitochondrial oxidation, resulting in high oxidative stress. ROS are constantly being generated in animals carrying out aerobic oxidation. ROS accompanied by poor antioxidant capacity results in oxidative stress [145]. Due to its lack of antioxidant enzymes, the pancreas is exceedingly vulnerable to the effects of oxidative stress, and the final consequence is the death of pancreas beta-cells, hence its contribution to the pathogenesis of diabetes [18, 145]. Kaempferol has been shown to improve cell viability and prevent pancreatic beta-cell apoptosis. When tested on cultured cells under high glucose concentrations, kaempferol promoted pancreatic beta-cell function and survival rate and insulin secretory function by restoring high-glucose-attenuated intracellular adenosine monophosphate (AMP) and adenosine triphosphate (ATP) production and improving the expression of anti-apoptotic genes *Akt* and *Bcl-2* [86]*.* Additionally, kaempferol tended to be inversely related to T2DM risk, and myricetin showed a protective role against T2DM in men and women across European countries in a large, prospective case–cohort study [177]. Other studies have supported that quercetin induces insulin secretion and protects beta-cell function and the pancreas from oxidative damage and inflammatory cytokines [173]. Quercetin also indirectly neutralizes oxidative stress via the activation of the nuclear erythroid 2-related factor 2-antioxidant response element (Nrf2-ARE) antioxidant pathway and stimulates the catalase and superoxide dismutase antioxidant enzymes [66]. Besides hyperglycemia, lipid peroxidation aggravates oxidative stress by converting the free fatty acids to free radicals via hydrogen extraction. Quercetin has proved to be an efficient antioxidant phytochemical because of its free radical scavenging activity and chelation of transition metal ions [34]. It is proposed that quercetin arrests lipid peroxidation by inhibiting xanthine oxidase, the enzyme responsible for catalyzing the oxidation of xanthine to uric acid and simultaneous superoxide formation [34, 84]. The mechanism describes more than the quercetin itself becomes a radical but too low in energy to become a deteriorating reactive one by the reaction with another free radical alongside the donation of a proton [52].

Some of the long-term diabetic complications are cataracts (diabetic retinopathy), neuropathy, and nephropathy because of sorbitol accumulation in the body. Quercetin exerts an inhibitory action on the aldose reductase enzyme, which catalyzes the conversion of glucose to sorbitol (a sugar alcohol moiety) [99].

The insulin resistance along with hyperglycemia causes inflammation, activating the body’s various inflammatory mechanisms with subsequent release of cytokines and inflammatory mediators, leading to an aberrant uncontrolled response known as “cytokine storm.” One consequence of this phenomenon is organ damage or, worse still, multiple organ damage failure. Quercetin possesses antioxidant and inhibitory effects on the inflammatory response because of its high-affinity interactions and inhibition of enzymes playing a role in oxidative reactions and processes, such as cyclooxygenase and lipoxygenase, following the inhibition of leukotrienes and prostaglandins (inflammatory mediators) [138, 145].

### Flavonols as antiviral agents

Flavonols may be considered an alternative treatment for various viral diseases due to the failure of conventional therapies to generate positive results. Experimental studies and in silico investigations have determined some promising therapeutic flavonols displaying the maximum antiviral effects [74, 101]. In this context, it is predicted that flavonols could become a consistent source of probable drugs and vaccines for various pandemic and epidemic diseases [150]. Even after the development of vaccines, severe acute respiratory syndrome coronavirus-2 (SARS-CoV-2) has still managed to halt the world due to the emergence of its various strains. Polyphenolic compounds stand out as potential treatment methods and future imminent biopharmaceuticals [3]. In SARS-CoV-2, the cytokine storm results from over-production of the pro-inflammatory cytokines due to the stimulation of the Nod-like receptor family and pyrin domain-containing 3 (NLRP3) inflammatory pathway. Myricetin, rutin, kaempferol, fisetin, and astragalin are among numerous other flavonols that display inhibitory effects on the synthesis and expression of the inflammatory mediators and cytokines in the prevention of the cytokine storm syndrome [57, 101, 111].

Quercetin suppresses NLRP3 inflammasome activation and inhibits lipopolysaccharide-induced production of various cytokines, including tumor necrosis factor-alpha (TNF-α), interleukin (IL)-6, IL-1β, and IL-8 in whole blood cells. Additionally, vitamin C and quercetin display a synergistic effect in combating viral infections [128]. The 3CL^pro^ protease is a vital enzyme of coronaviruses. It cleaves the polyproteins (pp1a and pp1ab) into the individual nonstructural proteins (Nsps) that form the RNA replicase–transcriptase complex, which mediates viral transcription and replication. Finally, virions are released from the infected host cells. Quercetin exhibited > 80% inhibition activity with an IC_50_ of 73 μM on the 3CL^pro^ protein product in vitro [31, 142]. The binding affinity of quercetin to the 3CL^pro^ protease enzyme is likely a critical factor in its antiviral activity. Quercetin is a type of flavonoid that has been shown to have antiviral properties against coronaviruses. Quercetin has been shown to inhibit the 3CL^pro^ protease enzyme of coronaviruses, which is essential for the cleavage the viral polyproteins into individual nonstructural proteins, enabling viral replication. The inhibition of this protease enzyme reduces viral replication and, hence, viral colonization load. The binding affinity of quercetin to the 3CL^pro^ protease is vital in determining its antiviral activity. A higher binding affinity between quercetin and the 3CL^pro^ protease can result in more robust inhibition of the enzyme’s activity, leading to more effective suppression of viral replication. Conversely, a lower binding affinity can result in weaker inhibition and may not be sufficient to control the virus’s spread.

Therefore, it can be inferred that the antiviral activity of quercetin is likely due to its ability to bind to the 3CL^pro^ enzyme with high affinity and inhibit its activity, thereby reducing viral replication and load. However, it is essential to note that the in vitro studies do not necessarily indicate the same antiviral activity in vivo, and further studies are required to determine the potential therapeutic use of quercetin for viral infections. Isoquercitrin (quercetin 3-*O*-glycoside [Q3G]) possessed inhibitory effects on the 3CL^pro^ of SARS-CoV [126]. Molecular modeling and docking studies asserted that Q3G interacts with Gln189, one of the key amino acid residues residing in the catalytic pocket of the 3CL^pro^ enzyme, arresting the 3CL^pro^ function [101]. Papain-like protease (PL^pro^) represents another therapeutic target for the pharmacophore because of its multifunctional roles during viral replication, specifically, its involvement in cleaving the N-terminal viral polyproteins to generate various Nsps (Nsp1, Nsp2, and Nsp3). The PL^pro^ is also used as a means of immune escape by the virus via inactivation of the interferon regulatory factor-3 (IRF3) pathway, resulting in reduced production of antiviral interferons, hence causing immunosuppression [106]. Many studies claim that quercetin has an excellent binding affinity with RNA-dependent RNA polymerase (RdRp), host cell receptor angiotensin-converting enzyme 2 (ACE2), spike protein, and PL^pro^ [70, 93]. Moreover, quercetin acts as a zinc ionophore, accelerating the transport of zinc ions across the lipid membranes. This action is crucial to blocking the entry of the virus into the host cells because coronavirus is vulnerable to the detrimental effects of zinc ions [3].

Acquired immunodeficiency syndrome (AIDS) is a chronic, potentially life-threatening health condition caused by the human immunodeficiency virus (HIV). The combined antiretroviral therapy (cART) introduced in 1996 constitutes several classes of drugs that act in concert to control and suppress HIV progression. The cornerstone of highly active antiretroviral therapy (HAART), which may also be called cART, is the co-administration of several different drugs that inhibit viral replication, an approach demonstrated to correlate with improved outcomes for patients with AIDS [80, 123, 135]. Nevertheless, the drugs pose long-term side effects. Moreover, emerging drug resistance, toxicity, lack of therapeutic effect, and restricted availability are among several disadvantages that have created avenues for the development of alternative medicines based on natural, medicinal compounds [122].

The HIV genome encodes for three enzymes essential for virus replication: protease (PR), reverse transcriptase (RT), and integrase (IN). The different subclasses and derivatives of flavonols act as multitarget agents by acting on these enzymes (PR, IN, and RT) while at the same time interfering and disrupting the steps in the viral replication process. The flavonol 7-*O*-glucoside herbacitrin, a kaempferol derivative with an additional hydroxyl group at the C-8 position, has shown such an effect, simultaneously inhibiting HIV-1 RT and HIV-IN. In an in vitro test designed to determine the potential target of herbacitrin, the data revealed marked inhibition of the HIV-1 RT at a high concentration of 21.5 μM herbacitrin compared to the inhibition of HIV-IN, which occurred at 2.15 μM [122]. RT was the first protein to be exploited for anti-HIV therapy. This multifunctional enzyme catalyzes the steps in the early stages of the viral replication process. It confers reverse transcriptase (RNA-dependent DNA polymerase, RDDP) activity, DNA-dependent DNA polymerase (DDDP), and the inherent ribonuclease H (RNase H) activity to catalyze the conversion of the viral genomic RNA into double-stranded DNA. Therefore, inhibition of each or any of the catalytic functions of RT will ultimately interfere with and disrupt the virus life cycle. Several quercetin- and kaempferol-based flavonol glycosides isolated from the leaves of *Thevetia peruviana* displayed notable inhibitory activity against RDDP, with IC_50_ of 20–43 μM, albeit the quercetin derivatives showing more activity than the kaempferol derivatives [150]. Kaempferol has displayed inhibiting effects on the RDDP of HIV-1 RT [112].

Furthermore, some flavonols block viral entry into the host cell by affecting the CD4 receptor and the CXCR4 and CCR5 co-receptors, eliminating the communication between the receptor and the cell’s internal pathways. In the TZM-bl cell line, a frequently used HIV-1 reporter cell line developed from HeLa cells, myricetin presented potent anti-HIV-1 activity, with > 87% inhibition of the infection and 49% inhibition against HIV-RT [112]. Thus, according to the World Health Organization (WHO) regulatory guidelines, myricetin has exhibited two crucial requirements to be developed as a microbicide: low toxicity and inhibitory activity against HIV infection [112].

### Antibacterial activity of flavonols

Pathogenic bacteria have become a threat to human beings. Before the first effective antibiotic, penicillin was disclosed in 1928, followed by sulfonamide medications in 1930, bacterial infections were the cardinal cause of death. As such, antibiotics have played a key role in managing contagious bacterial infections over the last 60 years [50]. However, the latest antibiotics reservoir has been slowly depleting since the 1970s, with a rising prevalence of antibiotic-resistant microorganisms [136], leading to a prominent post-antibiotic age [8]. Nevertheless, it is anticipated that plant-derived flavonols will play a vital role in combating bacteria and may prove to be an alternative drug treatment against bacteria (Table 2).

**Table 2:** Flavonols and their inhibitory dose concentrations against several types of bacteria

Flavonol	Susceptible bacteria	Function	Dose concentration (mcg/mL)	Reference
Kaempferol	*Helicobacter pylori*	Bactericidal effect	1000	[102]
Quercetin	*Pseudomonas aeruginosa*	Antibiofilm activity	20	[107], [108], [123]
	*Shigella flexneri*	No visible effect	500	
	*Lactobacillus casei* var.* shirota*	No visible effect	500	
	*Proteus vulgaris*	Damage to cell walls and membranes by enhancing alkaline phosphatase and β-galactosidase production	300	
	*Escherichia coli*	Damage to cell walls and membranes by enhancing alkaline phosphatase and β-galactosidase production	400	
	*Staphylococcus aureus*	Bacteriostatic effect via > 70% inhibition of biofilm formation	20	
Fisetin	*Streptococcus suis*	Target suilysin to hinder apertures and hemolysis	32	[116]

Kaempferol displays a dose-dependent inhibitory action against *Helicobacter pylori,* the bacterium responsible for human gastric cancer and peptic ulcer diseases worldwide [41]. *Helicobacter pylori* is a coil-shaped, neutralophilic, Gram-negative bacterium that inhabits the human bowel*. Helicobacter pylori* infection constitutes the predominant risk factor for gastric cancer and peptic ulcers combined, representing over a million annual fatalities [10, 104]. Combined treatments include proton-pump inhibitors and antibiotics (tetracycline, amoxicillin, clarithromycin, or metronidazole) [107]. Nevertheless, because of the high incidence of resistance to antibiotics, *H. pylori* elimination may not always be effective. According to a contemporary analysis in South American countries, antibiotic resistance trends differ dramatically by medication and region [21]. Kaempferol substantially inhibited two *H. pylori* strains (26,695 and 43,504) at 1000 mcg/mL in a liquid culture medium [41]. Moreover, a synergistic effect was observed after the combined application of kaempferol and (−)-epicatechin.

The omnipresent flavonol quercetin is considered to have antibacterial properties. *Escherichia coli* (Gram-negative bacterium) and *Staphylococcus aureus* (Gram-positive bacterium) were used to assess the antibacterial function of quercetin [157]. Gram-positive bacteria do not possess an outer lipopolysaccharide cell wall but harbor a thick peptidoglycan cell wall membrane. By contrast, Gram-negative bacteria have an outer lipopolysaccharide cell wall but a relatively thin peptidoglycan cell wall membrane. Quercetin damaged the cell walls and membranes of both *E. coli* and *S. aureus* via ameliorating the activity of the alkaline phosphatase and β-galactosidase enzyme concentrations in vitro, an effect that increased exponentially with increasing quercetin amounts [157]. It was also observed that quercetin had a greater bacteriostatic effect against Gram-positive bacteria than Gram-negative bacteria when tested against *E. coli*, P*seudomonas* aeruginosa, S*almonella* enterica Typhimurium, and S. aureus [157]. In another study, quercetin was tested for its antibacterial activity against *P. aeruginosa*, *Shigella flexneri*, *Lactobacillus casei* var *shirota*, *Proteus vulgaris*, *E. coli*, and *S. aureus* [67]. The minimum inhibitory concentrations of quercetin were 20 mcg/mL against S. aureus and P. aeruginosa, 300 mcg/mL against P. vulgaris, and 400 mcg/mL against E. coli [67, 161]. However, S*.* flexneri and L*.* casei var shirota were unaffected, even at 500 mcg/mL of quercetin [67].

Quercetin displays antiquorum sensing potential against *P. aeruginosa*. Research demonstrated the in vitro cytoprotective activity of quercetin against *P. aeruginosa* infection in human embryonic kidney cells (HEK 293 T), revealing 100% inhibition of the tested isolates by quercetin at 500 μg/mL, besides notable virulence attenuation and biofilm formation inhibition while also protecting the host epithelial cells [156]. These effects were observed even at a concentrated dose of 10,000 mcg/mL of quercetin because quercetin does not have major adverse effects on normal human cells [33, 120, 156]. In another example, four *P. aeruginosa* isolates (YU-V10, YU-V11, YU-V15, and YU-V28) with resistance to ciprofloxacin, gentamicin, amikacin, imipenem, and ceftazidime antibiotics were collected from urinary catheters to assess the antibiofilm efficacy of quercetin using the reference strain *P. aeruginosa* (PAO1), which was sensitive to all the tested antibiotics [156]. At a concentration of 250 mcg/mL, quercetin suppressed the biofilm formation of all isolates by about 43% to 79% compared with the reference strain. An initial determination indicated that although quercetin did not inhibit the growth of PAO1, it markedly inhibited biofilm formation by up to 51% at 8–64 mcg/mL and exerted a dramatic negative effect on the virulence factors [108]. Other studies have also supported the antibiofilm efficacy of quercetin [154, 178].

*Streptococcus suis* serotype 2 infection is a dangerous, porcine pathogen and zoonotic agent that has captured global attention since the first human case was reported in Denmark in 1968. Several virulence determinants are involved in the pathogenesis of the infection caused by *S. suis* serotype 2. New strategies for finding antivirulence molecules that can kill or hinder zoonotic bacterial infections have been published in recent decades. Fisetin was revealed as a potent antagonist to *S. suis-*mediated pathogenesis, specifically by suppressing the hemolytic activity of suilysin, a critical virulence factor [181]. To infect the host, *S. suis* must subvert epithelial blockades, evade the host’s immune system, replicate in the blood circulation, and infiltrate multiple organs, ultimately causing necrosis of the tissues, cells, or organs [43, 51]. The *S. suis* serotype 2 strain SC19 is known to secrete suilysin encoded by the *sly* gene and is highly noxious to pigs and mice [168, 179]. Suilysin exhibits antiphagocytic and antibactericidal properties in response to neutrophils and macrophages [12, 23, 43]. To achieve the maximal inhibition of suilysin in vitro, 32 mcg/mL of fisetin was required [181]. In vivo, 0.1 mcg/kg of fisetin showed therapeutic activity in SC19-infected mice, decreasing the bacterial loads and pro-inflammatory ability and improving the survival rate [181].

### Other pharmaceuticals properties of flavonols

#### Antioxidant agents

Flavonols are a form of flavonoid that has been widely researched for their antioxidant qualities. Antioxidants are substances that protect cells from free radical damage, which is generated by highly reactive molecules in the body that create oxidative stress. Flavonols have been discovered to be powerful antioxidants capable of protecting cells from oxidative stress and lowering the risk of chronic illnesses such as cancer, heart disease, and diabetes [134]. Flavonols such as quercetin, kaempferol, and myricetin have been proved in vitro to effectively scavenge free radicals such as superoxide anion, hydroxyl radical, and peroxyl radical [69, 125]. Flavonols can also bind metal ions, which can limit free radical generation. Metal ions such as iron and copper can stimulate the generation of free radicals, which can be prevented by chelating them with flavonols [28, 125]. Flavonols can help renew antioxidants such as vitamin C and vitamin E in the body [125]. Vitamins C and E are key antioxidants that may protect cells from oxidative stress, and flavonols have been shown to boost their levels in the body while also improving their antioxidant activity [81, 105]. Flavonols can also stimulate the production of antioxidant enzymes such as superoxide dismutase (SOD) and catalase in the body [22, 81]. These enzymes are critical in preventing oxidative stress and lowering the risk of chronic illnesses.

#### Anti-neurodegenerative agents

Several studies have shown that flavonols benefit brain health, especially in preventing and treating neurodegenerative diseases like Alzheimer’s and Parkinson’s [72, 100]. Flavonols have strong antioxidant qualities that can help protect brain cells from oxidative stress, which has been linked to the onset and progression of neurodegenerative disorders [7, 40]. Flavonols’ anti-inflammatory qualities can aid in the reduction of inflammation in the brain [97]. The flavonol fisetin was discovered to lower brain inflammation and enhance cognitive performance [97]. Kaempferol has neuroprotective properties and might be utilized to protect neurons from injury [171]. As a result, it was discovered to preserve neurons in the brain and increase cognitive performance. By enhancing the production of brain-derived neurotrophic factor (BDNF), a protein involved in the development and survival of neurons, quercetin was discovered to increase spatial memory and learning [140]. Flavonols have anti-neurodegenerative properties through acting as antioxidants, lowering inflammation, providing neuroprotection, and boosting memory and learning.

#### Anti-inflammatory agents

Flavonols are being investigated extensively for their possible anti-inflammatory effects. Flavonols contain a number of anti-inflammatory qualities, including the ability to block pro-inflammatory enzymes, reduce cytokine synthesis, modulate immune cell activity, and inhibit the NF-B pathway. Flavonols have been shown to reduce the activity of pro-inflammatory enzymes such cyclooxygenase (COX) and lipoxygenase (LOX). These enzymes are essential in the synthesis of inflammatory mediators such as prostaglandins and leukotrienes. Flavonols like quercetin and kaempferol have been demonstrated to decrease COX and LOX activity, which can lower inflammatory mediator production and relieve inflammation [47]. Flavonols have also been shown to inhibit the production of pro-inflammatory cytokines including tumor necrosis factor-alpha (TNF-) and interleukin-6 (IL-6) [162]. These cytokines are important in the development of chronic inflammation, and inhibiting them with flavonols can help reduce inflammation [37, 98]. Flavonols have been proved to influence the activity of inflammatory immune cells such as macrophages and T lymphocytes [109]. Flavonols like quercetin and kaempferol can block macrophage activation, which reduces the generation of pro-inflammatory mediators. Flavonols can also affect T cell activity, which is important in the progression of chronic inflammation [109]. The nuclear factor-kappa B (NF-kB) pathway is an important inflammatory regulator, and its activation can result in the creation of pro-inflammatory mediators [155]. Flavonols like quercetin and kaempferol have been demonstrated to block the NF-B pathway, reducing the generation of pro-inflammatory mediators and alleviating inflammation [155].

#### Anti-osteoporotic agents

Flavonols can promote bone mineralization by stimulating osteoblasts, the cells responsible for bone production [124]. In research, flavonol quercetin boosted bone density and structure by promoting bone production [166]. Furthermore, Flavonols have the ability to suppress osteoclasts, the cells responsible for bone resorption, and hence prevent bone loss [124]. In research, flavonol kaempferol was discovered to decrease bone resorption and enhance the bone density by decreasing osteoclast activity. Quercetin’s anti-inflammatory effects decrease bone tissue inflammation and help prevent bone loss caused by estrogen shortage [124, 166]. Myricetin’s anti-oxidative property performs the same thing by lowering oxidative stress [146]. However, further study is needed to discover the appropriate amount and duration of flavonol supplementation in people to prevent and cure osteoporosis.

#### Hepatoprotective agents

Flavonoids have also been shown to have potential hepatoprotective properties and may help to prevent or treat liver damage and disease. The liver is an important organ that aids in detoxification and metabolism, and it is vulnerable to harm from many poisons and medicines. Silymarin, a flavonoid derived from milk thistle, has been shown to have potent hepatoprotective effects by reducing oxidative stress, inflammation, and liver fibrosis [2, 102]. Flavonols are powerful antioxidants that may scavenge free radicals and protect liver cells from oxidative damage [81]. Oxidative stress is a key source of liver damage and inflammation, and flavonols like quercetin and kaempferol have been shown in animal experiments to protect against oxidative stress-induced liver damage [36, 109, 152]. Chronic inflammation can contribute to the development of liver disease, and flavonols have been discovered to have anti-inflammatory characteristics that can aid in the relief of liver inflammation [6]. Flavonols like quercetin and apigenin have been shown in animal experiments to decrease liver inflammation by blocking the generation of pro-inflammatory cytokines and the activation of inflammatory cells [176]. Liver fibrosis is a frequent complication of chronic liver illness and is defined by the buildup of extracellular matrix proteins, which can compromise liver function. Flavonols with antifibrotic characteristics [11, 40, 134], such as quercetin and catechin, have been discovered to limit the deposition of extracellular matrix proteins and diminish liver fibrosis. Flavonols have been discovered to modulate lipid metabolism and enhance lipid profiles, which can contribute to the development of fatty liver disease. Flavonols such as quercetin and kaempferol have been shown to minimize liver fat buildup and enhance lipid profiles by influencing the expression of genes involved in lipid metabolism [6].

#### Antimalarial agents

Recent studies supported that several flavonols have potential applications regarding antimalarial response. Quercetin and kaempferol are the most extensively studied for their antimalarial properties [7, 119, 134]. Research showed they could inhibit the growth of *Plasmodium falciparum* (malaria parasite) both in vivo and in vitro [45, 115]. In addition, fisetin with quercetin and kaempferol are individually capable of inhibiting the formation of hemozoin [45, 115, 119, 182, 183] resulting in the parasite’s death. Moreover, quercetin, fisetin, and kaempferol’s antioxidant and anti-inflammatory roles were proved beneficial for malaria treatments. Another flavonol named myricetin has been proved to suppress *Plasmodium falciparum* development in vitro [146]. Myricetin accomplishes this by impairing the parasite’s capacity to use glucose, which is critical for survival. Furthermore, myricetin possesses antioxidant and anti-inflammatory characteristics that may be useful in the treatment of malaria [143]. Rutin is a flavonoid glycoside that has been proved to have antimalarial properties. Rutin does this by reducing the development of the *Plasmodium berghei* malaria parasite in vivo [5, 113]. Rutin does not appear to influence hemozoin production, although its antimalarial mechanism is unknown.

#### Future perspectives of Flavonols

Flavonols are a kind of flavonoid that has been intensively researched for their possible health advantages. Flavonols have been demonstrated in recent study to have a wide variety of uses, including the prevention and treatment of chronic illnesses.

Studies have shown flavonols to enhance endothelial function, decrease inflammation, and lower blood pressure, all of which are vital factors in preventing cardiovascular disease[7, 134]. Furthermore, flavonols may help prevent and manage type 2 diabetes by enhancing insulin sensitivity and decreasing inflammation [40]. New flavonol derivatives with better drug activity and pharmacokinetic characteristics have been developed thanks to advances in chemical synthesis and structural modification methods [113, 115]. These new flavonols may have the ability to overcome current medication limitations such as drug resistance and toxicity. Another interesting application for flavonols is cancer prevention and therapy [131]. Flavonols have been found in recent research to decrease cancer cell proliferation and trigger death in cancer cells, making them a promising choice for cancer therapy. Combining flavonols with other anticancer medicines may increase effectiveness and decrease drug resistance. This method might be employed more widely in the future to increase the efficacy of flavonols as anticancer medicines [39, 78, 151]. Furthermore, flavonols may have chemopreventive characteristics, lowering the risk of cancer development.

Finally, flavonols have been proved to have neuroprotective properties as well as the potential to boost cognitive performance [72, 100]. According to recent study, flavonols can improve memory, attention, and executive function, making them a possible therapy for neurodegenerative disorders like Alzheimer’s [72, 100]. Furthermore, flavonols may help protect the brain from oxidative stress and inflammation-related damage [78]. Flavonols may be used in conjunction with other antimalarial medications as an adjuvant treatment to minimize inflammation and oxidative stress associated with the condition [45, 115].

## Conclusions

In the medicinal world, many drug compounds have significant mechanistic properties against various diseases. Most of these compounds are naturally acquired and have anticancer, antiviral, and antibacterial properties. Quercetin, myricetin, kaempferol, fisetin, rutin, and astragalin are examples of flavonols with high functionality, among other phytochemicals. Kaempferol, quercetin, fisetin, and myricetin show anticancer activity, and kaempferol is effective against *H. pylori*, a bacterium responsible for human gastric cancer. In addition, quercetin acts as an antioxidant that inhibits many enzymes, and kaempferol improves cell viability and prevents pancreatic beta-cell apoptosis. Kaempferol can also improve the function, survival rate, and insulin secretion of pancreatic beta-cells, supporting its antidiabetic properties.

Moreover, some flavonols block viral entry into the host cells and inhibit viral replication. There is also evidence that plant-derived flavonols might play a vital role in combating bacteria, providing alternative drug treatment. Based on these findings, we can conclude that flavonols have diverse biological properties of medicinal importance against various notorious diseases.

## Data Availability

All data are available within this manuscript.

## Abbreviations

UV: Ultraviolet
ROS: Reactive oxygen species
MYB: Myeloblastosis
AR: Androgen receptor
SGLT1: Sodium-glucose cotransporter 1
GLUT2: Glucose transporter 2
3CL^pro^: 3C-like protease
Sp1: Specificity protein 1
PI3K/Akt: Phosphoinositide 3-kinase/Ak strain transforming
PSA: Prostate-specific antigen
HK2: Human protein kallikrein 2
LNCaP: Lymph node carcinoma of the prostate
ERK: Extracellular signal-regulated kinase
Bcl-2: B-cell lymphoma-2
MMP: Mitochondrial membrane potential
LLC-MK2: Lilly Laboratories Culture-Monkey Kidney 2
MMP-2: Matrix metalloprotein-2
AP-1: Activator protein-1
EGFR: Epidermal growth factor receptor
T1DM: Type 1 diabetes mellitus
AMP: Adenosine monophosphate
ATP: Adenosine triphosphate
Nrf2-ARE: Nuclear erythroid 2-related factor 2-antioxidant response element
SARS-CoV-2: Severe acute respiratory syndrome coronavirus-2
NLRP3: Nod-like receptor family and pyrin domain-containing 3
Nsps: Nonstructural proteins
PL^pro^: Papain-like protease
RdRp: RNA-dependent RNA polymerase
ACE2: Angiotensin-converting enzyme 2
RDDP: RNA-dependent DNA polymerase
HEK: Human embryonic kidney cells
